# Environmental scanning electron microscopy analysis of *Proteus mirabilis* biofilms grown on chitin and stainless steel

**DOI:** 10.1007/s13213-014-0978-9

**Published:** 2014-09-20

**Authors:** Milagro Fernández-Delgado, Zoilabet Duque, Héctor Rojas, Paula Suárez, Monica Contreras, María A. García-Amado, Carlos Alciaturi

**Affiliations:** 1 Centro de Biofísica y Bioquímica, Instituto Venezolano de Investigaciones Científicas, Caracas, Venezuela; 2 Unidad de Biodeterioro Industrial, Fundación Instituto Zuliano de Investigaciones Tecnológicas, La Cañada, Estado Zulia Venezuela; 3 Instituto de Inmunología, Facultad de Medicina, Universidad Central de Venezuela, Caracas, Venezuela; 4 Departamento de Biología de Organismos, Universidad Simón Bolívar, Caracas, Venezuela

**Keywords:** *Proteus mirabilis*, Adhesion, Biofilms, Chitin, Stainless steel, Environmental scanning electron microscopy

## Abstract

*Proteus mirabilis* is a human pathogen able to form biofilms on the surface of urinary catheters. Little is known about *P. mirabilis* biofilms on natural or industrial surfaces and the potential consequences for these settings. The main aim of this work was to assess and compare the adhesion and biofilm formation of *P. mirabilis* strains from different origins on chitin and stainless steel surfaces within 4 to 96 h. Using environmental scanning electron microscopy, the biofilms of a clinical strain grown on chitin at 4 h showed greater adhesion, aggregation, thickness, and extracellular matrix production than those grown on stainless steel, whereas biofilms of an environmental strain had less aggregation on both surfaces. Biofilms of both *P. mirabilis* strains developed different structures on chitin, such as pillars, mushrooms, channels, and crystalline-like precipitates between 24 and 96 h, in contrast with flat-layer biofilms produced on stainless steel. Significant differences (*p* < 0.05) were found in the frequency of pillars and channels. Images of transmission electron microscopy demonstrated abundant fimbriae in 100 % of cells from both strains, which could be related to surface adherence and biofilm formation. This represents the first study of *P. mirabilis* showing adhesion, biofilm formation, and development of different structures on surfaces found outside the human host.

## Introduction

*Proteus mirabilis* is a Gram-negative bacterium and an important human pathogen commonly isolated from urinary tract infections (UTIs) such as those occurring in catheterized patients or individuals with structural abnormalities of the urinary tract (Warren et al. 1982). This bacterium carries numerous virulence factors that are important for causing UTIs, including bacterial adhesion to the uroepithelium mediated by fimbriae (Manos and Belas 2006), which also contribute to the localization of bacteria in the bladder, and to biofilm formation (Jacobsen and Shirtliff 2011). Besides UTI, the pathogen has been described as an etiological agent of diverse opportunistic and nosocomial infections of the respiratory tract and of wounds, burns, skin, eyes, ears, nose, and throat, as well as in gastroenteritis resulting from the intake of contaminated food (Muller 1989; Rozalski et al. 1997; Manos and Belas 2006). Additionally, it is known to be widely distributed in the environment, occurring in water (Ajayi and Akonai 2003), manure, soil (Manos and Belas 2006), and oysters (Fernández-Delgado et al. 2007), where it has been isolated showing multiple antibiotic resistance with potential public health risks (Ajayi and Akonai 2003; Fernández-Delgado et al. 2007).

In most natural, clinical, or industrial settings, bacteria are found predominantly as irreversibly adhered communities or so-called biofilms, rather than planktonic cells. Biofilms have been defined in the literature as “microorganisms attached to a surface and covered with exopolysaccharides (EPS) of microbial origin” (Charackerlis and Marshall 1990). Biofilm formation is a development process, which initially involves the adhesion of bacterial cells to a surface and production of EPS resulting in more firmly and irreversible bacterial attachment that cover and protect the cells from adverse conditions (Davey and O'Toole 2000). Maturation of biofilm architecture begins displaying, in many species such as *P. mirabilis*, structures that have been observed to resemble a “mushroom-like” arrangement with nutrient channels interspersed within them (Jones et al. 2007). Finally, detachment and dispersion of the cells from the biofilms occur to colonize new areas with a transition from a sessile to a planktonic phenotype (Davey and O’Toole 2000).

Substratum material is one of the factors affecting the bacterial adhesion and growth of biofilms (Azevedo et al. 2006). In relation to the substratum origin, organic and inorganic surfaces have been shown to influence the attachment and biofilm formation of particular pathogens (Azevedo et al. 2006; Pruzzo et al. 2008). In the aquatic environment, *Vibrio cholerae* and *Helicobacter pylori* have been reported to be associated and forming biofilms with a variety of organic surfaces, including chitinous animals (e.g. shrimp, zooplancton, crabs), bivalves (oysters), and aquatic plants (Cellini et al. 2005; Pruzzo et al. 2008), as well as inorganic substrates of anthropogenic origin such as stainless steel, copper, glass, polyvinyl chloride, polypropylene, and polystyrene (Azevedo et al. 2006; Cellini et al. 2008). Among these materials, chitin is the second most abundant biopolymer in nature after cellulose, and comprises an important source of organic carbon and nitrogen (Pruzzo et al. 2008). Chemically, chitin is regarded as a fairly intractable material since it is insoluble in most ordinary solvents such as water, alcohols, acetone, hexane, diluted acids, and diluted and concentrated alkalis (Bough et al. 1978). On other side, stainless steel possesses high surface free energy which determines its hydrophilic nature and the bacterial attachment (Kokare et al. 2009). Stainless steel is widely used in food processing equipment because of its high resistance to corrosion by food products and detergents, and it has been demonstrated to be highly hygienic and to fulfill the regulations for the alimentary processing industry (Jullien et al. 2002). With respect to substratum structure, microbial colonization enhances as surface roughness increases due to the higher surface area (Kokare et al. 2009).

A number of microscopy techniques have been developed aimed at a deeper understanding about the composition, properties, and function of biofilm formation. Scanning electron microscopy (SEM) and confocal scanning laser microscopy have been used to observe the structure of *P. mirabilis* biofilm grown in conditions that mimic urinary catheters (Jones et al. 2007; Schlapp et al. 2011; Holling et al. 2014a, b). However, preparation by SEM may mechanically disturb delicate samples, compromise morphological information, or introduce other artefacts (Collins et al. 1993). On the contrary, environmental scanning electron microscopy (ESEM), a special variant of SEM or environmental mode, has the potential to render biofilm images without the dehydration process that could potentially alter the structure of biofilms due to the control of the water vapour pressure inside the microscope. This makes it possible to visualize biofilm surfaces in the wet, native state with a minimum of sample damage and changes in specimen morphology (Schwartz et al. 2009; Holling et al. 2014a, b).

Is well known that *P. mirabilis* forms biofilms on the surface of urinary catheters (Jones et al. 2007; Schlapp et al. 2011). Besides urinary tract surfaces, little is known about its ability to adhere on natural or industrial surfaces, and the only existing, available data of *P. mirabilis* isolation from biofilms formed on stainless steel surfaces are from an ice cream plant (Gunduz and Tuncel 2006). Therefore, the work described here aimed to assess and compare by ESEM the adhesion and biofilm formation of *P. mirabilis* strains from clinical and environmental origins on two different surfaces, the chitin found abundantly in invertebrate organisms and aquatic environments, and the stainless steel used in industrial processes, ship hulls, drinking water distribution systems, and clinical instruments, as other potential sites for biofilm formations.

## Materials and methods

### Bacterial strains

The clinical strain of *P. mirabilis* used in this study was obtained from the Centro Venezolano de Colecciones de Microorganismos (CVCM 620, Caracas, Venezuela), an isolate from an encrusted indwelling urethral catheter. The environmental strain *P. mirabilis* M was isolated from oyster samples (*Isognomon alatus*) collected at the northwestern coast of Venezuela and identified by biochemical and molecular methods (Fernández-Delgado et al. 2007). Both strains were cultured in MacConkey (Difco, Detroit, MI, USA) at 37 °C for 24–48 h and stored at −80 °C in Nutrient Broth (Difco, Detroit, MI, USA) media supplemented with 15 % (v/v) glycerol until their use.

### Observation by transmission electron microscopy (TEM) of *P. mirabilis* morphological features

Cellular morphology of *P. mirabilis* strains from clinical and environmental origins was observed by TEM and the frequency of their appendages was determined in the total observed cells. The strains were grown in Luria-Bertani broth (LB, Sigma-Aldrich, Munich, Germany) at 37 °C in logarithmic phase. The cells were harvested by centrifugation at 327 *g* (Eppendorf, Hamburg, Germany) for 10 min, resuspended in 10 μmol l^−1^ Tris–HCl buffer at pH 7.4 and negatively stained with 2 % aqueous uranyl acetate. The specimens were examined on a transmission electron microscope model FEI CM10 (FEI, Eindhoven, Holland) at an accelerating voltage of 80 kV.

### Test surfaces and bacterial suspensions for in vitro biofilms

Coupons measuring 1 cm^2^ were prepared from shrimp exuviae (source of chitin) and stainless steel as surfaces of adhesion. The chitin coupons were washed three times with sterile distilled water and heated for 24 h at 60 °C. The stainless steel coupons were immersed in dichloromethane (Sigma-Aldrich, Munich, Germany) and subsequently in 100 % ethanol with gentle sonication (5–10 min of ultrasound at 30 % of amplitude, 750 W, 20 kHz, in an Ultrasonic Processor, Cole-Parmer Instruments, Vernon Hills, IL, USA). Coupons were finally placed in vials containing 4.5 ml of nutrient broth and autoclaved for 15 min at 121 °C.

*Proteus mirabilis* strains were grown until the beginning of the logarithmic phase in nutrient broth at 37 °C. After incubation, the total cell concentration for clinical (1.00 × 10^5^ cells ml^−1^) and environmental (1.29 × 10^5^ cells ml^−1^) strains was obtained using the LIVE/DEAD BacLight kit (Molecular Probes, Eugene, OR, USA) and by counting the viable and dead cells.

### In vitro biofilm assays

Aliquots of 0.5 ml of *P. mirabilis* cultures in nutrient broth were inoculated to vials with media to expose test coupons. Cultures were incubated at 37 °C for 4, 24, 48, and 96 h by quadruplicate. Vials containing media and coupons without inocula were included as controls. After each incubation time, coupons from each *P. mirabilis* culture were removed, rinsed three times with sodium cacodylate buffer (0.1 mol l^−1^, pH 7.4), immediately immersed in 2.5 % glutaraldehyde, and kept at 4 °C until ESEM analysis.

### Biofilm observation by ESEM

Environmental scanning electron micrographs were obtained using a Quanta 200FEG ESEM (FEI Company, Eindhoven, the Netherlands) operated at an acceleration voltage of 4–6 kV and a chamber pressure of about 130 to 190 Pa. Duplicate samples of chitin and stainless steel coupons cultured with *P. mirabilis* strains were glued to the holders using die-cut carbon conductive adhesive discs (SPI Supplies/Structure Probe, Inc., West Chester, PA, USA). About 20 different measurement positions on the surface of each sample were chosen randomly to obtain representative images over biofilm formation. We compared frequencies of the different structures formed in the biofilms of clinical and environmental *P. mirabilis* strains on chitin surface between 24 and 96 h, by counting the number of structures in each frame. The non-parametric Mann–Whitney test for two independent samples was employed to analyze these differences (*p* < 0.05).

## Results

### Morphological differences of *P. mirabilis* strains analysed by TEM

**Fig. 1:**
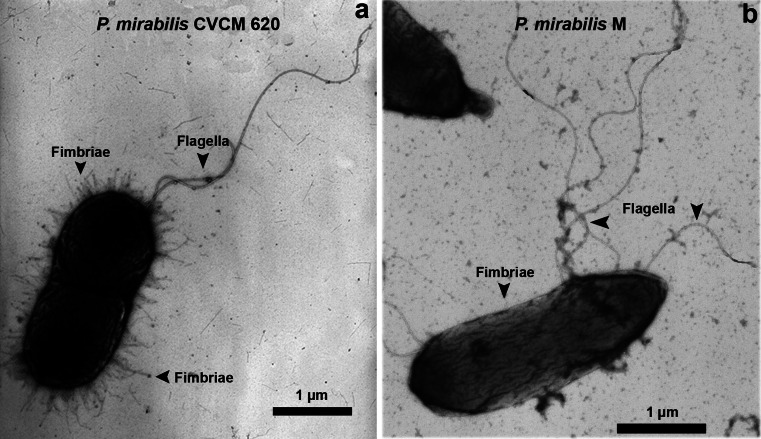
Representative electron micrographs of cells positively stained of clinical **a** and environmental **b** *P. mirabilis* strains. Notice fimbriae and flagella in both strains

*Proteus mirabilis* strains were observed by TEM as rod-shaped with fimbriae in 100 % of cells. The clinical and environmental strains showed multiple flagella in 56.1 % (Fig. 1a) and 12.3 % of the observed cells (Fig. 1b), respectively. Pili were scarce and found in similar proportions for the clinical (12.1 %) and environmental (15.4 %) strains. Fimbriae could be distinguished from flagella by their shorter and finer appearance (Fig. 1) whereas pili were similar in structure to fimbriae, but longer.

### Adhesion and biofilm formation of *P. mirabilis* evaluated by ESEM

**Fig. 2:**
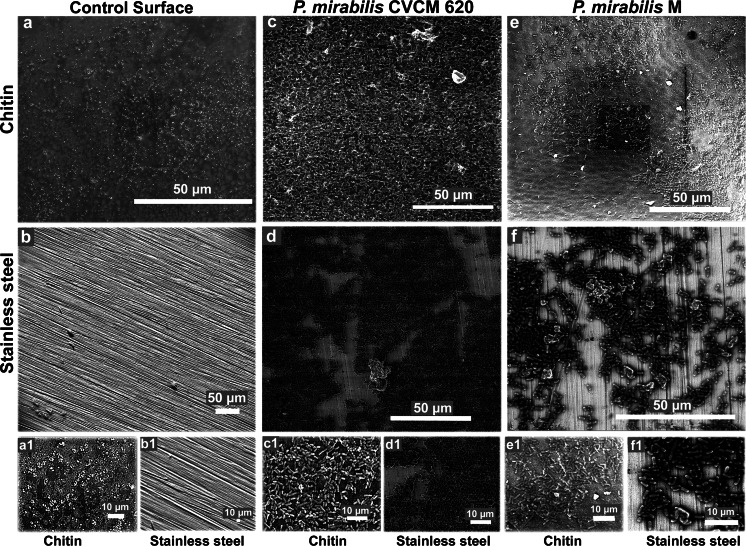
Representative ESEM images of control surfaces tested without bacterial inoculation and *P. mirabilis* biofilms formed at 4 h: **a** chitin and **b** stainless steel control surfaces. Clinical *P. mirabilis* biofilms formed on chitin **c** and stainless steel **d**. Environmental *P. mirabilis* biofilms formed on chitin **e** and stainless steel **f**. Details of original images **a**, **b**, **c**, **d**, **e**, and **f** are shown in the lower images **a1**, **b1**, **c1**, **d1**, **e1,** and **f1**, respectively

ESEM analysis of *P. mirabilis* biofilms formed on chitin and stainless steel surfaces revealed substantial differences over time (4–96 h). Fig. 2 compares the biofilms of clinical and environmental *P. mirabilis* grown on chitin or stainless steel at 4 h. Figure 2a, b shows the two surfaces tested, chitin and stainless steel, respectively, without bacterial inoculation. Chitin disclosed a heterogeneous rough surface (Fig. 2a and 2a1) while the stainless steel showed homogeneous and parallel grooves (Fig. 2b and 2b1). Clinical and environmental *P. mirabilis* cells were observed attached to the chitin and stainless steel surfaces at 4 h, indicating that both surfaces were rapidly and readily colonized when compared to control surfaces. At this time point, the biofilms of the clinical strain grew in the whole area of chitin with extensive aggregation and extracellular matrix production (Fig. 2c and 2c1), while in the stainless steel biofilms had slower growth with absence of bacteria and extracellular matrix in some areas of the surface (Fig. 2d and 2d1). Likewise, environmental *P. mirabilis* biofilms formed on chitin (Fig. 2e and 2e1) were more confluent with production of extracellular matrix than those grown on the stainless steel surface (Fig. 2f and 2f1). These last images (Fig. 2f and 2f1) were obtained at a higher magnification than Fig. 2e and 2e1 to show the difference better. In summary, there was a higher growth of the clinical strain compared to the environmental strain on chitin and stainless steel, showing that the biofilms developed a better cell arrangement on chitin.

**Fig. 3:**
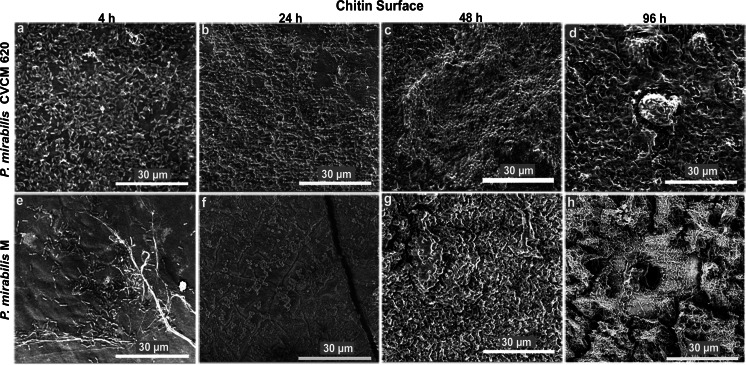
Representative ESEM images of biofilms formed by clinical **a-d** and environmental **e**-**h** *P. mirabilis* strains on chitin during the period from 4 to 96 h, showing an evolution panel from thin layer biofilms to highly organized biofilms

Since chitin showed more growth for *P. mirabilis* biofilms than stainless steel surfaces, this study thoroughly compared the biofilm development of clinical and environmental strains on the chitin surface in time during the period of 4 to 96 h, showing an evolution panel from a thin layer to highly organized biofilms (Fig. 3). Initially (4 h), there was an abundant growth of the clinical strain with a homogeneous distribution in the field, while the environmental strain displayed a lesser growth with an irregular distribution (Fig. 3a and e). At 24 h, the clinical strain showed dense biofilms and incipient structures covered with an extracellular matrix (Fig. 3b), whereas the environmental strain showed mosaic-like flat biofilms with broken areas similar to channels (Fig. 3f). At 48 h, the growth of the clinical and environmental *P. mirabilis* evolved to more complex biofilms with prominent formations similar to peaks and valleys in the clinical strain (Fig. 3c) and coarse biofilms with obvious channels in the environmental one (Fig. 3g). In the last period (96 h), both strains showed architecture in the biofilms with developed structures embedded in the extracellular matrix, such as high formations (Fig. 3d) and tunnels surrounded by many channels (Fig. 3h).

**Fig. 4:**
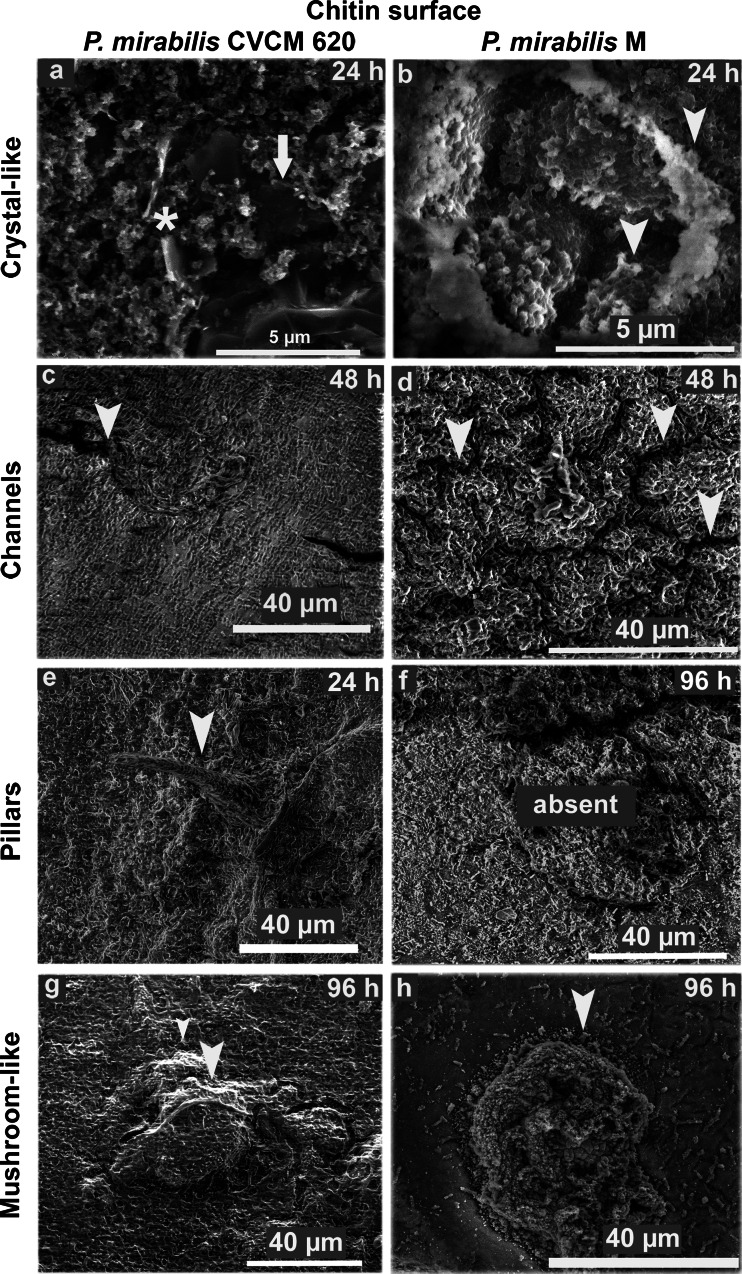
Representative ESEM images of structures formed by *P. mirabilis* on chitin between 24 and 96 h. Crystalline-like depositions observed at 24 h on the clinical strain as type 1 (*) and type 2 (*arrow*) crystals **a**, and on the environmental strain as saturated amorphous crystalline material **b**. Channels interspersed within biofilms of clinical **c** and environmental **d** strains at 48 h. Pillar formations present at 24 h and absent until 96 h in the clinical **e** and environmental **f** strains, respectively. Mushroom-like structures with channels embedded in the middle of the biofilm of clinical and environmental strains at 96 h **g** and **h**, respectively. Head arrows indicate the structures in each case of study

**Fig. 5:**
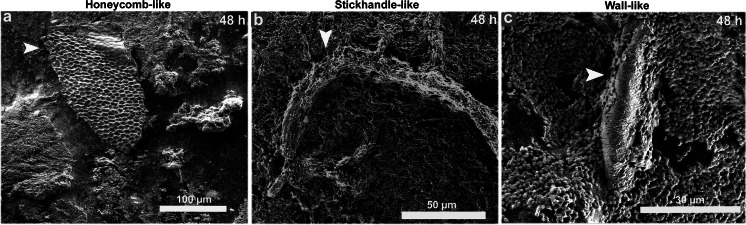
Representative ESEM images of other highly organized structures formed by the *P. mirabilis* clinical strain on chitin at 48 h. Structures like honeycombs **a**, stick handles **b**, and walls **c**. Head arrows indicate the structures in each study case

During the period from 24 to 96 h, four structures typically reported in the literature were evident on the chitin: crystalline-like precipitates, channels, pillars, and mushrooms. Crystalline-like depositions attached to the surface and to the bacterial cells were observed in both strains at 24 h (Fig. 4a and b). Two distinct crystal types were found embedded within biofilms of the clinical strain: large electron-dense structures known as type 1 crystals (Fig. 4a, *) and flat ‘sheet-like’ structures, a recent description of crystals observed by ESEM in *P. mirabilis* catheter biofilms named type 2 (Fig. 4a, arrow) by Holling et al. (2014a, b). With respect to the environmental strain, some areas of the biofilms were saturated with amorphous crystalline material without well-defined crystal structures (Fig. 4b). Low numbers of channels were observed within the biofilm for the clinical strain at 48 h (Fig. 4c) when compared with their high frequency in the environmental strain biofilms at the same time (Fig. 4d), resulting in a significant difference (*p* < 0.05; Table 1). Elevated formations with the characteristic architecture of pillars reported for *V. cholerae* (Watnick and Kolter 1999) were present in the biofilms of the clinical strain at 24 h (Fig. 4e), but absent in the biofilms of the environmental strain until the last period of the study (96 h), where this difference was statistically significant (*p* < 0.05; Table 1). On the other side, mushroom-like structures commonly reported in the literature to *P. mirabilis* biofilms with flow channels embedded and interspersed within them were found at 96 h in the biofilms of clinical and environmental strains (Fig. 4g and h), with a prominent cellular growth surrounding the structure for the clinical strain. Other highly organized structures not previously described were seen during 24 to 96 h only in the biofilm of the clinical strain, and these were like honeycombs, stick handles, and walls. Representative images are shown in Fig. 5 at 48 h.

**Table 1:** Frequency of structures observed by frames in biofilms of *P. mirabilis* strains formed on chitin between 24 and 96 h. S, number of frames with structure observed; F, number of frames. The non-parametric Mann–Whitney test for two independents samples indicated significant differences for pillars and channels *p < 0.05

Structure	Clinical strain (CVCM 620)	Environmental strain (M)
	S/F (%)	S/F (%)
Pillars	8/18 (44.4)*	0/22 (0.00)*
Channels	2/14 (14.3)*	16/21 (76.2)*
Mushrooms	5/17 (29.4)	7/22 (31.8)
Crystalline-like precipitates	16/33 (48.5)	8/30 (26.7)

S, number of frames with structure observed; F, number of frames; *p < 0.05

## Discussion

In this research, we evaluated and compared the biofilm formation of clinical and environmental *P. mirabilis* strains using two different organic (chitin) and inorganic surfaces (stainless steel), commonly found in natural, industrial, or clinical systems. Initially, our interest focused on the morphological differences among the distinct strains employed in the biofilm study. By TEM analysis, we found predominant fimbriae in both *P. mirabilis* strains (100 % of observed cells) and less frequency of flagella for the environmental (12.3 %) compared to the clinical (56.1 %) cells. Fimbriae have been described as one of the most important virulence factors for this pathogen in the attachment to the uroepithelial cell surfaces and colonization of the surrounding tissues (Manos and Belas 2006). On other hand, flagella play an important role in a multicellular spinning movement described for this bacterium (Liu et al. 2014) and have been shown to contribute positively to the first stage of biofilm formation, accelerating the attachment to the surface despite the fact that they are not required for this process. Additionally, flagellar motility has a role in surface perception, triggers the swarmer cell differentiation, and participates in the migration and ascending process (Jacobsen and Shirtliff 2011). Flagellar mutants of *P. mirabilis* are deficient in in vitro catheter-associated biofilm formation and also exhibit attenuated host urinary tract colonization (Jones et al. 2004). In our study, the fewer number of flagella may be the cause of the flat biofilms displayed by the environmental strain of *P. mirabilis* in respect to the higher frequency of flagella and biofilm with greater organized and elevated structures of the clinical strain.

The use of ESEM in this study allowed the visualization of *P. mirabilis* biofilms fully hydrated and in their native state (McGregor and Donald 2010). Our ESEM observations have shown for the first time that strains of *P. mirabilis* from different origins are able to form contrasting types of biofilm structures on chitin and stainless steel surfaces. Chitin contained larger numbers of *P. mirabilis* adhered cells and aggregates from any origin in a few hours (4 h; Fig. 2), and biofilm architecture from 24 to 96 h (Fig. 3), when compared to the stainless steel surface (data not shown). In these biofilms, it was typical to see complex and highly organized structures consisting of pillars, channels, and “mushroom” shapes embedded in abundant matrix and crystalline-like precipitates. Pillars of bacteria attached to chitin have been reported for *V. cholerae* in mature biofilms and constitute a successful environmental survival mechanism that significantly influences the lifestyle of the bacterium (Pruzzo et al. 2008). This new biofilm architecture exhibited by the clinical strain of *P. mirabilis* could enable the bacterium to survive in an ecosystem which demands major challenges with respect to the urinary niche. On the other hand, the mushroom-like structure has been previously observed in mature biofilms of clinical strains of *P. mirabilis* and *Pseudomonas aeruginosa* at 24 h (Costerton et al. 1999; Jones et al. 2007). In the “mushroom” biofilm, the colonies are arranged in a regular self-repeating manner allowing flow between clusters, and hence, convective transport of nutrients (Eberl et al. 2000). The structure of these microbial biofilms can also vary in response to environmental conditions such as nutrient limitation, flow rate, shear, and pressure (Stoodley et al. 2002). Similarly, in our study, the clinical and environmental strains of *P. mirabilis* displayed mushroom-like biofilms surrounded by channels, with the last ones more pronounced and in higher and significant frequency in the environmental strain than the clinical one. This might be due to the need to increase the flow of water and nutrients inside the biofilm as a different strategy of survival in exposure to the aquatic environmental conditions. Crystalline-like biofilms of *P. mirabilis* have been well described to block urinary catheters due to deposition of crystals within these structures where it can contribute to renal damage and can frequently cause complications, such as stone formation in the kidneys and bladder as well as bacteremia (Manos and Belas 2006). Recent studies conducted by Holling et al. (2014a, b) have evaluated by ESEM *P. mirabilis* crystalline biofilms in situ on urinary catheters, revealing two types of crystals in the mature biofilm (Holling et al. 2014a) and providing new insights on the mechanisms involved in their formation (Holling et al. 2014b). Similarly, the clinical strain of our study showed two crystal types immersed in the bulk biofilm matrix which looked like to large electron-dense (type 1 crystal) and flat "sheet-like" (type 2 crystal) structures. Both crystals were absent in the environmental strain biofilms, characterized by saturated, amorphous crystalline material. A possible explanation for the absence of type 1 and type 2 crystals in the environmental strain could be based on a different expression of the urease activity, reported in *P. mirabilis* to generate alkaline conditions and mineralization of the biofilm (Stickler and Hughes 1999). Additionally, mutants of this pathogen with an attenuated ability to form crystalline structures showed less expansive biofilms with a more uniform topology and fewer type 2 crystals (Holling et al. 2014a). The characteristic biofilms formed by the environmental strain agreed with these previous observations and indicate that this strain could mimic a mutant or defective strain in the aquatic niche. There are no reports in the literature concerning *P. mirabilis* crystalline biofilms of environmental origin, suggesting the potential of a marine strain to developed crystalline biofilms. Other structures were seen in the clinical strain biofilms resembling honeycombs, stick handles, and walls. To our knowledge, these structures have not been previously described and could be related to survival or colonization strategies in a natural surface such as the chitin.

In contrast to chitin, smaller aggregates of clinical and environmental *P. mirabilis* were ubiquitous on the stainless steel surface at 4 h (Fig. 2), and flat layer biofilms could be observed between 24 and 96 h (data not shown). Our results suggest that the organic composition and surface roughness of chitin could influence the greater bacterial attachment and biofilm development. A chitin-binding protein (*chb*) has been identified in the transcriptome of swarming cells of *P. mirabilis* (Pearson et al. 2010). It is widely known that surface proteins play a role in the attachment of other bacteria to chitin, such as *Vibrio* spp., allowing its association with chitinous fauna and providing a number of advantages, mainly food availability, adaptation to environmental nutrient gradients, tolerance to stress, and protection from toxic compounds and predators (Pruzzo et al. 2008). Probably, *P. mirabilis* has developed a similar ability to attach to chitin and to survive in the environment.

In conclusion, this study has shown for first time the ability of clinical and environmental *P. mirabilis* strains to develop contrasting biofilms on chitin and stainless steel surfaces. The best biofilm formation was found on chitin suggesting a successful bacteria-substrate interaction. Our results provide important insights to the current knowledge of *P. mirabilis* biofilms, expanding their potential sites outside the human host.

## References

[CR1] Ajayi AO, Akonai KA (2003). Antibiotic sensitivity profile of microorganisms encountered in the Lagos Lagoon, Nigeria. Afr J Biomed Res.

[CR2] Azevedo NF, Pacheco AP, Keevil CW, Vieira MJ (2006). Adhesion of water stressed *Helicobacter pylori* to abiotic surfaces. J Appl Microbiol.

[CR3] Bough WA, Salter WL, Wu ACM, Perkin BE (1978). Influence of manufacturing variables on the characteristics and effectiveness of chitosan products. I. Chemical composition, viscosity, and molecular-weight distribution of chitosan products. Biotechnol Bioeng.

[CR4] Cellini L, Di Campli E, Grande R, Di Bartolomeo S, Prenna M, Pasquantonio MS, Pane L (2005). Detection of *Helicobacter pylori* associated with zooplankton. Aquat Microb Ecol.

[CR5] Cellini L, Grande R, Di Campli E, Di Bartolomeo S, Di Giulio M, Traini T, Trubiani O (2008). Characterization of an *Helicobacter pylori* environmental strain. J Appl Microbiol.

[CR6] Charackerlis WG, Marshall KC, Charackerlis WG, Marshall KC (1990). Biofilms: a basis for an interdisciplinary approach. Biofilms.

[CR7] Collins SP, Pope RK, Scheetz RW, Ray RI, Wagner PA, Little BJ (1993). Advantages of environmental scanning electron microscopy in studies of microorganisms. Microsc Res Tech.

[CR8] Costerton JW, Stewart PS, Greenberg EP (1999). Bacterial biofilms: a common cause of persistent infections. Science.

[CR9] Davey ME, O’toole GA (2000). Microbial biofilms: from ecology to molecular genetics. Microbiol Mol Biol Rev.

[CR10] Eberl HJ, Picioreanu C, Heijnen JJ, van Loosdrecht MCM (2000). A three-dimensional numerical study on the correlation of spatial structure, hydrodynamic conditions, and mass transfer and conversion in biofilms. Chem Eng Sci.

[CR11] Fernández-Delgado M, Contreras M, García-Amado MA, Gueneau P, Suárez P (2007). Occurrence of *Proteus mirabilis* associated with two species of Venezuelan oysters. Rev Inst Med Trop Sao Paulo.

[CR12] Gunduz G, Tuncel G (2006). Biofilm formation in an ice cream plant. Antoine van Leeuwenhoek.

[CR13] Holling N, Dedi C, Jones CE, Hawthorne JA, Hanlon GW, Salvage JP, Patel BA, Barnes LM, Jones BV (2014). Evaluation of environmental scanning electron microscopy for analysis of *Proteus mirabilis* crystalline biofilms in situ on urinary catheters. FEMS Microbiol Lett.

[CR14] Holling N, Lednor D, Tsang S, Bissell A, Campbell L, Nzakizwanayo J, Dedi C, Hawthorne JA, Hanlon G, Ogilvie LA, Salvage JP, Patel BA, Barnes LM, Jones BV (2014). b) Elucidating the genetic basis of crystalline biofilms formation in *Proteus mirabilis*. Infect Immun.

[CR15] Jacobsen SM, Shirtliff ME (2011). *Proteus mirabilis* biofilms and catheter-associated urinary tract infections. Virulence.

[CR16] Jones BV, Young R, Mahenthiralingam E, Stickler DJ (2004). Ultrastructure of *Proteus mirabilis* swarmer cell rafts and role of swarming in catheter-associated urinary tract infection. Infect Immun.

[CR17] Jones SM, Yerly J, Hu Y, Ceri H, Martinuzzi R (2007). Structure of *Proteus mirabilis* biofilms grown in artificial urine and standard laboratory media. FEMS Microbiol Lett.

[CR18] Jullien C, Bénézech T, Carpentier B, Lebret V, Faille C (2002). Identification of surface characteristics relevant to the hygienic status of stainless steel for the food industry. J Food Eng.

[CR19] Kokare CR, Chakraborty S, Khopade AN, Mahadik KR (2009). Biofilm: Importance and applications. Indian J Biotechnol.

[CR20] Liu Y, Deng Y, Luo S, Deng Y, Guo L, Xu W, Liu L, Liu J (2014). Observation of multicellular spinning behavior of *Proteus mirabilis* by atomic force microscopy and multifunctional microscopy. Micron.

[CR21] Manos J, Belas R, Dworkin M (2006). The Genera *Proteus*, *Providencia* and *Morganella*. The Prokaryotes.

[CR22] McGregor JE, Donald AM (2010). ESEM imaging of dynamic biological processes: the closure of stomatal pores. J Microsc.

[CR23] Muller HE (1989). The role of Proteae in diarrhea. Zbl Bakt.

[CR24] Pearson MM, Rasko DA, Smith SN, Mobley HL (2010). Transcriptome of swarming *Proteus mirabilis*. Infect Immun.

[CR25] Pruzzo C, Vezzulli L, Colwell RR (2008). Global impact of *Vibrio cholerae* interactions with chitin. Environ Microbiol.

[CR26] Rozalski A, Sidorczyk Z, Kotelko K (1997). Potential virulence factors of *Proteus* bacilli. Microbiol Mol Biol Rev.

[CR27] Schlapp G, Scavone P, Zunino P, Härtel S (2011). Development of 3D architecture of uropathogenic *Proteus mirabilis* batch culture biofilms-A quantitative confocal microscopy approach. J Microbiol Meth.

[CR28] Schwartz T, Jungfer C, Heibler S, Friedrich F, Faubel W, Obst U (2009). Combined use of molecular biology taxonomy, Raman spectrometry, and ESEM imaging to study natural biofilms grown on filter materials at waterworks. Chemosphere.

[CR29] Stickler D, Hughes G (1999). Ability of *Proteus mirabilis* to swarm over urethral catheters. Eur J Clin Microbiol Infect Dis.

[CR30] Stoodley P, Cargo R, Rupp CJ, Wilson S, Klapper I (2002) Biofilm material properties as related to shear-induced deformation and detachment phenomena. J Ind Microbiol Biotechnol 29:361–367 10.1038/sj.jim.7000282 12483479

[CR31] Warren J, Tenney JH, Hoopes JM, Kass EH (1982). A prospective microbiologic study of bacteriuria in patients with chronic indwelling urethral catheters. J Infect Dis.

[CR32] Watnick PI, Kolter R (1999). Steps in the development of a *Vibrio cholerae* El Tor biofilm. Mol Microbiol.

